# Disparities in Spatial Access to Emergency Surgical Services in the US

**DOI:** 10.1001/jamahealthforum.2022.3633

**Published:** 2022-10-14

**Authors:** Marta L. McCrum, Neng Wan, Jiuying Han, Steven L. Lizotte, Joshua J. Horns

**Affiliations:** 1 Division of General Surgery, University of Utah, Salt Lake City; 2 Department of Geography, University of Utah, Salt Lake City; 3 Surgical Population Analysis Research Core, Department of Surgery, University of Utah, Salt Lake City

## Abstract

**Importance:**

Hospitals with emergency surgical services provide essential care for a wide range of time-sensitive diseases. Commonly used measures of spatial access, such as distance or travel time, have been shown to underestimate disparities compared with more comprehensive metrics.

**Objective:**

To examine population-level differences in spatial access to hospitals with emergency surgical capability across the US using enhanced 2-step floating catchment (E2SFCA) methods.

**Design, Setting, and Participants:**

A cross-sectional study using the 2015 American Community Survey data. National census block group (CBG) data on community characteristics were paired with geographic coordinates of hospitals with emergency departments and inpatient surgical services, and hospitals with advanced clinical resources were identified. Spatial access was measured using the spatial access ratio (SPAR), an E2SFCA method that captures distance to hospital, population demand, and hospital capacity. Small area analyses were conducted to assess both the population with low access to care and community characteristics associated with low spatial access. Data analysis occurred from February 2021 to July 2022.

**Main Outcomes and Measures:**

Low spatial access was defined by SPAR greater than 1.0 SD below the national mean (SPAR <0.3).

**Results:**

In the 217 663 CBGs (median [IQR] age for CBGs, 39.7 [33.7-46.3] years), there were 3853 hospitals with emergency surgical capabilities and 1066 (27.7%) with advanced clinical resources. Of 320 million residents, 30.8 million (9.6%) experienced low access to any hospital with emergency surgical services, and 82.6 million (25.8%) to advanced-resource centers. Insurance status was associated with low access to care across all settings (public insurance: adjusted rate ratio [aRR], 1.21; 95% CI, 1.12-1.25; uninsured aRR, 1.58; 95% CI, 1.52-1.64). In micropolitan and rural areas, high-share (>75th percentile) Hispanic and other (Asian; American Indian, Alaska Native, or Pacific Islander; and 2 or more racial and ethnic minority groups) communities were also associated with low access. Similar patterns were seen in access to advanced-resource hospitals, but with more pronounced racial and ethnic disparities.

**Conclusions and Relevance:**

In this cross-sectional study of access to surgical care, nearly 1 in 10 US residents experienced low spatial access to any hospital with emergency surgical services, and 1 in 4 had low access to hospitals with advanced clinical resources. Communities with high rates of uninsured or publicly insured residents and racial and ethnic minority communities in micropolitan and rural areas experienced the greatest risk of limited access to emergency surgical care. These findings support the use of E2SFCA models in identifying areas with low spatial access to surgical care and in guiding health system development.

## Key Points

**Question:**

How does spatial access to emergency surgical services vary across the US, and what community characteristics are associated with low access to care?

**Findings:**

In this cross-sectional study using advanced geospatial metrics that capture distance, hospital capacity, and population demand for all 320 million US residents in 2015, an estimated 1 in 10 residents experienced low access to any hospital with emergency surgical capabilities, and 1 in 4 experienced low access to hospitals with advanced clinical resources. Communities with high proportions of uninsured, publicly insured, and racial and ethnic minority groups in micropolitan and rural regions were at the greatest risk of being in low-access areas.

**Meaning:**

Substantial disparities exist in spatial access to emergency surgical care across the US; comprehensive metrics of spatial access, such as enhanced 2-step floating catchment models, should be adopted to identify targets for surgical health system development.

## Teaser

This cross-sectional study estimates differences in access to hospitals with emergency surgical capability across the US using advanced geospatial metrics that capture distance, hospital capacity, and population demand.

## Introduction

Emergency general surgery (EGS) conditions constitute a growing public health burden in the US, accounting for more than 7% of inpatient admissions, greater than half of all surgical mortality, and more than $28 billion in health care costs annually.^1,2,3,4^ These time-sensitive surgical conditions range from common, low-risk diseases such as appendicitis to high-risk diseases such as gastrointestinal perforation that pose an immediate threat to life.^5^ Physical access to hospitals with surgical capabilities is critical to patients with EGS diseases, and as such, there is an urgent need to adopt comprehensive measures of spatial access to surgical care that can identify inadequately served communities and guide regional health system development.

Spatial access to care encompasses measures of both availability and accessibility.^6^ Availability includes the number of locations at which a patient may receive care, the capacity of the hospital to provide care, and nearby population-level demand. Accessibility captures the travel impedance between patients and hospitals, often measured in distance or travel time. Prior studies of spatial access to surgical care have predominantly focused on impedance measures, which are certainly important in the care of emergency conditions, but have represented a limited perspective of access. Importantly, they do not capture the accessibility of other hospitals in a region, nor the ability of hospital capacity to meet population demand, both of which are essential to determining whether a given system provides adequate spatial access to care. Enhanced 2-step floating catchment area (E2SFCA) models offer a possible solution to this problem by considering both supply-demand relationships (availability) and travel impedance (accessibility) and may offer additional valuable information about population-level disparities that can be underestimated when using more conventional means.^7,8,9^

We sought to apply advanced geospatial methods using E2SFCA models and small area analysis to examine population-level differences in spatial access to hospitals with emergency surgical capabilities across the US. We had 3 central goals: first, to evaluate spatial access to any EGS-capable hospital; second, to assess access to hospitals with advanced clinical resources only, because management of complicated or severe disease may necessitate resources primarily restricted to these centers; and third, to evaluate sociodemographic characteristics of communities at high risk for limited access to emergency surgical care. Because living in rural and micropolitan regions may heighten exposure to unequal social conditions that perpetuate disparities in access to health care and racially and ethnically minoritized communities in rural areas have been shown to experience disparities in access to health care and health insurance compared with White residents, we stratified our analysis by statistical area type (metropolitan, micropolitan, and rural).^10,11^ We hypothesized that substantial population disparities would exist in access to overall and advanced-resource hospitals that may exceed those previously reported and that racially and ethnically minoritized communities in rural and micropolitan locations would experience greater disparities in spatial access to surgical care compared with those in urban areas.

## Methods

This cross-sectional study followed the Strengthening the Reporting of Observational Studies in Epidemiology (STROBE) reporting guideline and was classified as nonhuman participant research by the policy of the University of Utah institutional review board.^12^ Informed consent was waived because census data are deidentified. Data analysis occurred from February 2021 to July 2022.

### Geospatial Data

We first constructed a geographic information science platform for EGS-capable hospitals in the US. Geospatial data for the US, including census units, zip code, city boundaries, and transportation networks, were obtained from the Census Topologically Integrated Geographic Encoding and Referencing file and the StreetMap North America network data set from the Environmental Systems Research Institute.^13,14^

### Hospital Identification

Hospital geographic location, clinical resources, and measures of utilization were obtained using the American Hospital Association (AHA) 2015 Annual Survey Database. We identified all nonfederal general service hospitals, then excluded limited-service hospitals, those that performed fewer than 10 inpatient surgical cases annually, and any hospital without emergency services. We then cross-referenced this cohort with the American College of Surgeons list of verified trauma centers to capture additional hospitals with emergency surgical services not included in the AHA Annual Survey Database (eFigure in the Supplement). Hospitals were classified as having “advanced resources” if they had at least 5 general intensive care unit beds (>25th percentile nationally), the presence of a computed tomography scanner, ultrasonography, and advanced gastroenterology services (identified by endoscopic retrograde cholangiopancreatography), or were in the top quartile of inpatient operations (>2753 procedures annually). These resources were selected for their relevance to management of EGS diseases and signaled a basic ability to care for patients with complex or severe illness. We also included hospitals verified as a level 1 or 2 trauma center, or those in the top quartile of inpatient surgical volume, to capture additional hospitals with advanced capabilities that did not report resources to the AHA survey. (See eTable 1 in the Supplement for hospital characteristics of nonadvanced and advanced-resource hospitals.)

### Spatial Access Measurement

We used the spatial access ratio (SPAR), an E2SFCA model, to measure spatial access to EGS-capable hospitals.^15,16,17^ Models using E2SFCA are a widely validated type of gravity model that incorporate both accessibility and availability of health services into a single measure of spatial access for a given population site.^7,8,9,18,19^ In brief, this method uses provider-to-population ratios (or hospital-to-population ratios) weighted by hospital capacity, potential patient volume, and travel impedance (eMethods in the Supplement).

To calculate SPAR, we used measures of supply, demand, and travel impedance. Supply, or hospital capacity, was measured using the number of inpatient hospital beds. Demand was approximated by the population of each census block group (CBG) and located at the population-weighted centroid. We set the catchment area to 60 minutes of driving time, as this represents a common benchmark for access to surgical care.^20,21,22,23,24^ Distance and travel time from population-weighted CBG centroid to hospital site were calculated using ArcMap software as described previously.

The measure of SPAR is presented as a ratio of the spatial access for the specific CBG relative to the national mean. Greater values of SPAR denote better spatial access, and SPAR values greater than 1 mean indicate better-than-national-average spatial access. Categories of SPAR were identified using Jenks natural breaks classification method, which seeks to reduce the variance within classes and maximize the variance between classes. We set 1 breakpoint at the national mean, as this was our reference point, then used the Jenks method to define categories above and below this point. We defined low access as the lowest 2 Jenks categories.^16^ We examined 3 discrete categories: SPAR greater than 1.0 (high access), SPAR within 0.3 to 1.0 (moderate access), and SPAR less than 0.3 (low access).

### Sociodemographic Characteristics

Sociodemographic characteristics examined included population size, race and ethnicity, poverty rate, health insurance coverage rate, and rural or urban status at the CBG level. Population, annual household income, and health insurance data were collected from 5-year estimates from the American Community Survey 2013 to 2017.^25^ Race and ethnicity were analyzed to evaluate the presence of racial and ethnic disparities in spatial access to care. Race and ethnicity were analyzed based on standard US Census Bureau categories with the reference as non-Hispanic white. Asian; American Indian, Alaska Native, or Pacific Islander; and 2 or more races and/or ethnicities were combined into a single category that we called “other racial and ethnic minority groups.” Census block groups were classified into racial and ethnic composition categories based on predominant patterns of residential segregation, including populations with greater than the 75th percentile non-Hispanic Black individuals, greater than the 75th percentile Hispanic individuals, and greater than the 75th percentile other racial and ethnic minority groups. Poverty was assessed using CBG median income and classified as below the federal poverty level (FPL), 100% to 200% of FPL, or greater than 200% FPL. Health insurance coverage was categorized as CBG residents with private employer-based insurance, public insurance (Medicare, Medicaid, Tricare/Veterans Administration or military-based insurance), or uninsured. The statistical area type of each CBG was derived from the census tract–level 2010 Rural Urban Commuting Area codes and categorized into 1 of 3 groups (metropolitan, micropolitan, and rural).

### Statistical Analysis

Sociodemographic disparities in spatial access were examined with CBG as the unit of analysis. Census block groups were excluded if they were missing information on rurality or sociodemographic factors of interest (n = 7101; 3.3%), which resulted in an analytic sample of 210 562 CBGs. We assessed differences in access within racial and ethnic minority and insurance categories by comparing high-share CBGs (defined as >75th percentile for the characteristic) with those less than the 75th percentile. Poverty was assessed by comparing the lower 2 income groups to CBGs with a median income of greater than 200% FPL. Demographics (including median race and ethnicity and insurance) are summarized and presented at the CBG level and are not population-weighted estimates.

We constructed multinomial regression models to test the associations among race and ethnicity, poverty, and insurance coverage with presence of low spatial access to EGS-capable hospitals at the CBG level. We ran univariable analyses of each cofactor individually. We then constructed multivariable regressions to model the association of census tract demographic characteristics, adjusting for median age, race and ethnicity, income, and health insurance coverage. Models were constructed for metropolitan, micropolitan, and rural areas separately. A sensitivity analysis was conducted to explore effect modification by introducing an interaction term between race and ethnicity and income to the aforementioned model, because evidence suggests that racial segregation and poverty can interact to intensify the effect of poverty.^26,27^ We were also concerned about the effect of spatial autocorrelation. Because there is not a readily available multinominal modeling strategy to account for spatial autocorrelation, we performed a second sensitivity analysis of low vs high access using mixed-effect logistic regression, with an exponential spatial covariance structure where the covariance between observations (CBGs) was based on the Euclidian distance between their centroids. The estimates for each demographic were only minimally changed, and the findings remain unchanged (eTable 6 in the Supplement). The level of significance assessed was *P* < .05. Analysis was completed using R statistical software, version 4.0.3 (R Foundation for Statistical Computing).

## Results

### Geospatial Access

**Figure 1.**
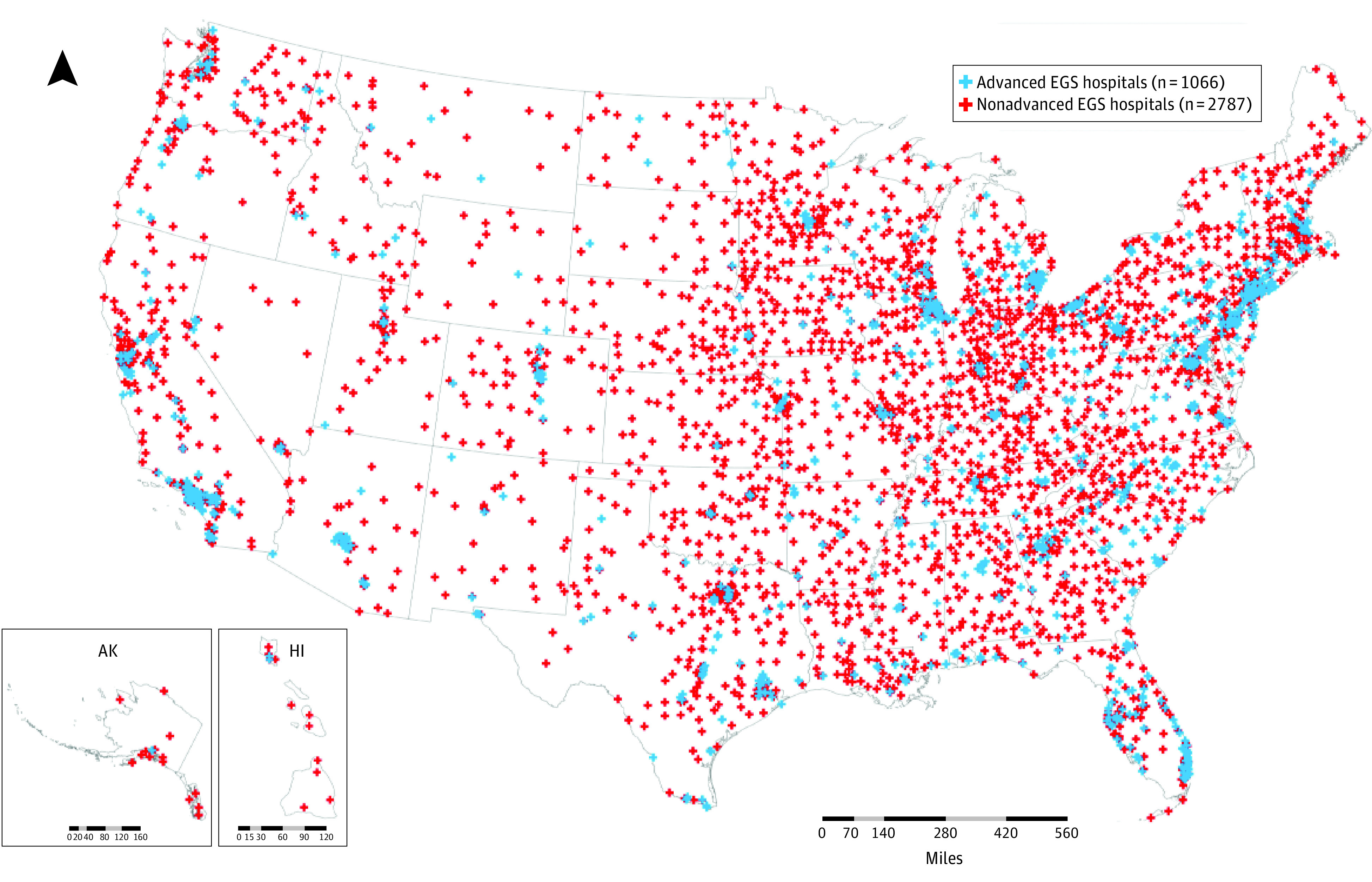
Spatial Distribution of Emergency General Surgery (EGS)–Capable Hospitals

**Figure 2.**
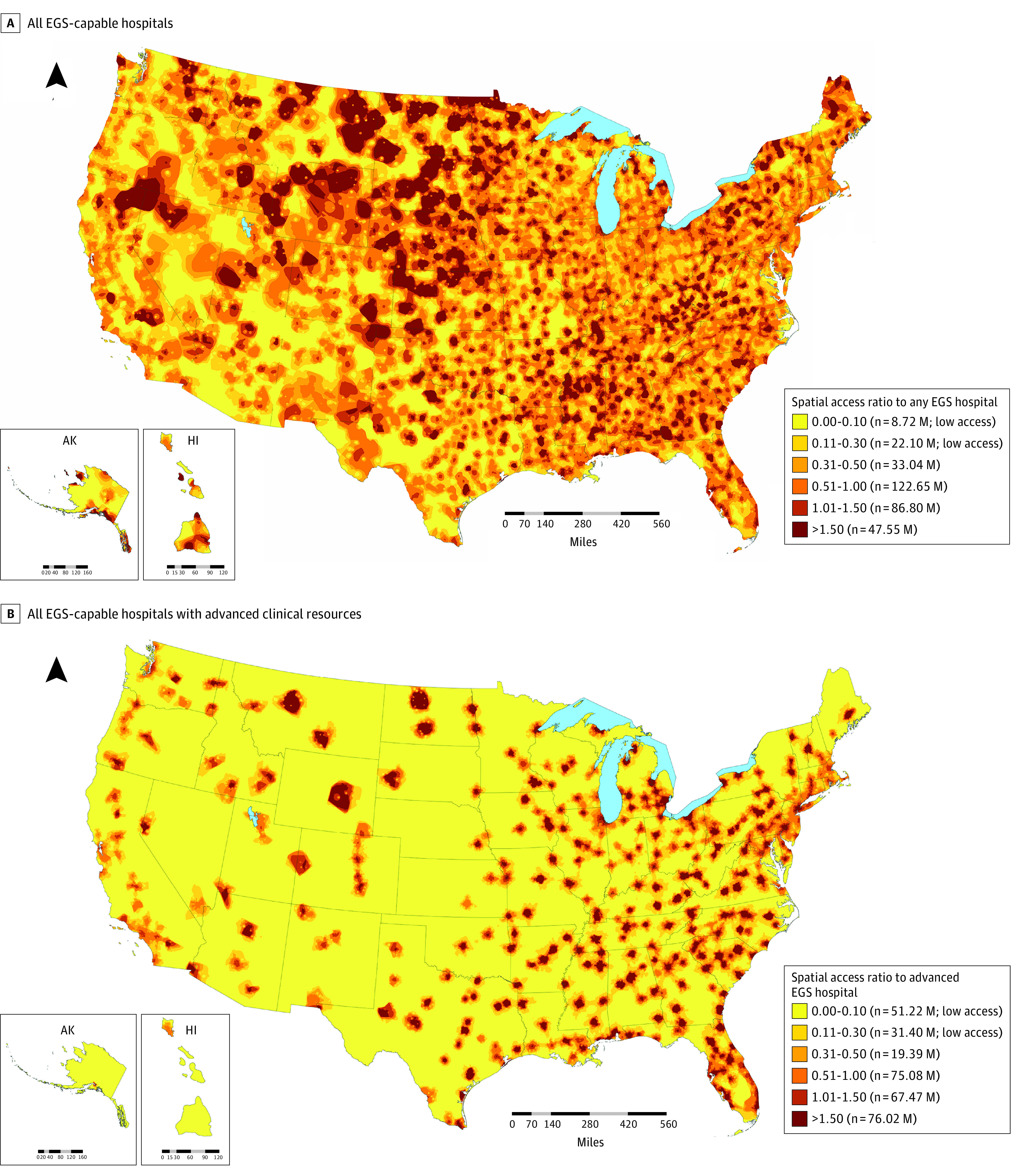
Spatial Access to Emergency General Surgery (EGS)–Capable Hospitals in the US Measured by Spatial Access Ratio

In the 217 663 CBGs (median [IQR] age for CBGs, 39.7 [33.7-46.3] years), 3853 hospitals with EGS capability were identified, of which 1066 (27.7%) were advanced-resource hospitals. Figure 1 shows the spatial distribution of all EGS hospitals and advanced EGS hospitals. All CBGs in the US were analyzed, with spatial access measured by SPAR to any EGS-capable hospital and advanced-resource hospital, shown in Figure 2. A total of 134.35 million people (42.0%) experienced high access (SPAR >1.0) to any EGS hospital, though 30.8 million (9.6%) experienced low access (SPAR <0.3) to EGS care, with 8.72 million (2.7%) in the lowest identified category (SPAR <0.1). When assessing access to hospitals with advanced clinical resources, disparities in spatial access grew, with 82.6 million people (25.8%) experiencing low access, and 51.22 million (16.0%) in the lowest category.

Regional analysis showed substantial variation in population within low-access areas. The Northeast had the lowest proportion with low access to any EGS hospital (4.6%), compared with the South (greatest, 11.2%). For advanced-resource hospitals, the Northeast again had the smallest percentage of low-access population (15.9%), and the Midwest the highest proportion (32.7%) (eTable 2 in the Supplement).

### Small Area Analysis of Disparities

A total of 210 562 CBGs were analyzed, representing 315 770 248 residents in the US. A total of 94 717 CBGs (45%) were high access, 93 952 (44.6%) were moderate access, and 21 893 (10.4%) were low access. Table 1 shows population characteristics of each access category. Compared with high-access CBGs, low-access CBGs had a higher median (IQR) age (44 [38-50] years vs 38 [33-45] years), lower proportion of Black, Hispanic, and other racial and ethnic minority designation, and higher proportion of nonpoor CBGs (57% vs 53%). Low-access areas had a higher proportion of rural CBGs (36%) vs high-access areas (5%); however, nearly half (49%) of low-access CBGs were classified as metropolitan areas.

**Table 1.** Census Block Group Characteristics

Characteristic	CBGs, No. (%)			
	Low access (SPAR <0.3) (n = 2189)	Medium access (SPAR 0.3-1) (n = 93 952)	High access (SPAR >1) (n = 94 717)	All (n = 210 562)
Age, median (IQR), y	43.7 (37.5-50.0)	40.0 (34.0-46.4)	38.3 (32.7-45.3)	39.7 (33.7-46.3)
Race and ethnicity, median (IQR), % population				
Black	0.2 (0-3.2)	1.6 (0-8.1)	5.6 (0.5-25.2)	2.5 (0-13.4)
Hispanic	2.7 (0.3-10.1)	7.4 (1.8-22.9)	5.9 (1.2-19.4)	6.1 (1.3-19.9)
White	89.7 (70.4-96.2)	75.7 (46.6-91.1)	66.3 (28.1-86.8)	73.7 (40.6-90.6)
Other racial and ethnic minority groups^a^	2.1 (0.4-5.0)	4.4 (1.3-10.9)	4.1 (1.2-9.8)	3.9 (1.0-9.7)
Race and ethnicity: high-share (>75th percentile) racial and ethnic minority CBG				
Black	2736 (12.5)	15 996 (17.0)	33 909 (35.8)	52 641 (25.0)
Hispanic	3179 (14.5)	26 277 (28.0)	23 185 (24.5)	52 641 (25.0)
Other racial and ethnic minority groups^a^	2505 (11.4)	26 359 (28.1)	23 772 (25.1)	52 636 (25.0)
Insurance status, median (IQR), % population				
Private insurance	55.9 (44.3-66.7)	6.27 (49-74)	57.1 (42-71)	59.6 (45-72)
Any public insurance, age 18-64 y	15.5 (8.9-25.0)	12.5 (6.2-22.0)	15.3 (7.2-27.0)	14.0 (6.9-25.0)
Uninsured	13.4 (7.3-22.0)	10.4 (4.7-19.0)	12.8 (6.0-23.0)	11.7 (5.5-21.0)
Insurance: high-share (>75th percentile) CBG				
Any public insurance, age 18-64 y	5588 (25.5)	19 085 (20.3)	27 968 (29.5)	52 641 (25.0)
Uninsured	5974 (27.3)	20 036 (21.3)	26 630 (28.1)	52 640 (25.0)
Poverty				
Nonpoor	12 570 (57.4)	65 687 (69.9)	50 137 (52.9)	128 394 (61.0)
Near poor (100%-200% FPL)	8603 (39.3)	25 417 (27.1)	36 130 (38.2)	70 150 (33.3)
Poor (below FPL)	720 (3.29)	2848 (3.03)	8450 (8.9)	12 018 (5.71)
Median income, $	51 842 (40 250-66 917)	62 321 (45 000-87 917)	50 417 (35 545-70 556)	55 357 (39 917-77 992)
Statistical area type				
Metropolitan	10 780 (49.2)	76 016 (80.9)	82 728 (87.3)	169 524 (80.5)
Micropolitan	3176 (14.5)	10339 (11.0)	7481 (7.9)	20 996 (10.0)
Rural	7937 (36.3)	7597 (8.1)	4508 (4.8)	20 042 (9.5)
Population density, median (IQR), persons/square mile	64.72 (20.09-313.89)	2224.04 (374.9-5428.25)	3862.94 (1515.21-8397.14)	2550.84 (409.35-6094.37)

Abbreviations: CBG, census block group; FPL, federal poverty level; SPAR, spatial access ratio.

^a^ The category “other racial and ethnic minority groups” comprised Asian; American Indian, Alaska Native, or Pacific Islander; or 2 or more races and/or ethnicities.

Table 2 shows the adjusted association between population characteristics and being in a low-access area for emergency surgical care. (Unadjusted analysis can be found in eTable 3 in the Supplement.) Insurance status was consistently associated with low access across all types of statistical areas. In metropolitan areas, CBGs with a high share of uninsured (adjusted rate ratio [aRR], 1.41; 95% CI, 1.33-1.49) and publicly insured residents (aRR, 1.07; 95% CI, 1.01-1.14) had increased risk of being low access. In micropolitan and rural areas, high share of Hispanic and other racial and ethnic minority residents was also associated with increased risk of low access to emergency surgical care, in addition to insurance status. (Adjusted analyses for all CBGs combined can be found in eTable 4 in the Supplement.)

**Table 2.** Multinomial Model of Factors Associated With Low-Access CBGs^a^

Characteristic	aRR (95% CI)			
	All EGS hospitals		Advanced clinical resource hospitals	
	Low access	Medium access	Low access	Medium access
**Metropolitan**				
Median age	1.03 (1.03-1.03)^b^	1.00 (1.00-1.00)^b^	1.01 (1.01-1.02)^b^	1.00 (1.00-1.01)^b^
High-share racial and ethnic minority community				
Black	0.33 (0.31-0.35)^b^	0.47 (0.46-0.49)^b^	0.39 (0.37-0.40)^b^	0.48 (0.47-0.50)^b^
Hispanic	0.63 (0.60-0.68)^b^	1.61 (1.57-1.65)^b^	0.61 (0.59-0.64)^b^	1.49 (1.45-1.53)^b^
Other racial and ethnic minority groups^c^	0.30 (0.28-0.32)^b^	1.13 (1.10-1.15)^b^	0.40 (0.39-0.42)^b^	1.27 (1.24-1.30)^b^
Median income				
Nonpoor (>200% FPL)	1 [Reference]	1 [Reference]	1 [Reference]	1 [Reference]
Near-poor (100%-200% FPL)	0.73 (0.69-0.77)^b^	0.53 (0.52-0.55)^b^	1.03 (0.99-1.07) (*P* = .10)	0.55 (0.53-0.57)^b^
Poor (below FPL)	0.23 (0.19-0.27)^b^	0.30 (0.29-0.33)^b^	0.58 (0.54-0.63)^b^	0.34 (0.32-0.36)^b^
High-share insurance group				
Public insurance	1.07 (1.01-1.14) (*P* = .02)	0.94 (0.91-0.96)^b^	1.17 (1.12-1.22)^b^	0.99 (0.96-1.02) (*P* = .41)
Uninsured	1.41 (1.33-1.49)^b^	0.84 (0.82-0.87)^b^	1.12 (1.07-1.16)^b^	0.81 (0.78-0.83)^b^
**Micropolitan**				
Median age	1.03 (1.03-1.04)^b^	1.01 (1.14-1.57)^b^	1.01 (1.00-1.02)^b^	1.00 (0.99-1.01) (*P* = .95)
High-share racial and ethnic minority community				
Black	0.51 (0.44-0.57)^b^	0.51 (0.47-0.56)^b^	0.47 (0.42-0.54)^b^	0.48 (0.4-0.58)^b^
Hispanic	1.35 (1.19-1.54)^b^	1.35 (1.23-1.48)^b^	2.95 (2.38-3.66)^b^	2.72 (2.10-3.53)^b^
Other racial and ethnic minority groups^c^	1.12 (0.99-1.28) (*P* = .07)	0.82 (0.75-0.90)^b^	1.76 (1.46-2.13)^b^	1.13 (0.88-1.46) (*P* = .34)
Median income				
Nonpoor (>200% FPL)	1 [Reference]	1 [Reference]	1 [Reference]	1 [Reference]
Near-poor (100%-200% FPL)	0.70 (0.63-0.77)^b^	0.82 (0.77-0.88)^b^	1.07 (0.95-1.20) (*P* = .28)	0.90 (0.77-1.06) (*P* = .21)
Poor (below FPL)	0.38 (0.31-0.47)^b^	0.57 (0.50-0.65)^b^	0.82 (0.66-1.02) (*P* = .07)	0.74 (0.55-1.01) (*P* = .21)
High-share insurance group				
Public insurance	1.34 (1.22-1.48)^b^	0.95 (0.88-1.02) (*P* = .14)	1.38 (1.22-1.57)^b^	1.28 (1.08-1.52) (*P* = .01)
Uninsured	1.40 (1.26-1.55)^b^	1.11 (1.03-1.20) (*P* = .01)	1.17 (1.03-1.33) (*P* = .02)	1.15 (0.96-1.37) (*P* = .13)
**Rural**				
Median age	1.03 (1.03-1.03)^b^	1.00 (1.00-1.01)	1.03 (1.02-1.04)^b^	1.00 (0.99-1.02) (*P* = .82)
High-share racial and ethnic minority community				
Black	0.64 (0.58-0.72)^b^	0.90 (0.80-0.99) (*P* = .06)	0.54 (0.41-0.72)^b^	0.80 (0.57-1.13) (*P* = .20)
Hispanic	1.80 (1.57-2.01)^b^	1.21 (1.05-1.40) (*P* = .01)	1.70 (1.08-2.68) (*P* = .02)	1.44 (0.85-2.42) (*P* = .18)
Other racial and ethnic minority groups^c^	1.31 (1.16-1.48)^b^	0.88 (0.78-1.01) (*P* = .06)	1.69 (1.09-2.61) (*P* = .02)	1.14 (0.69-1.90) (*P* = .61)
Median income				
Nonpoor (>200% FPL)	1 [Reference]	1 [Reference]	1 [Reference]	1 [Reference]
Near-poor (100%-200% FPL)	0.84 (0.78-0.92)^b^	0.87 (0.80-0.95) (*P* = .01)	1.06 (0.83-1.35) (*P* = .67)	0.87 (0.65-1.17) (*P* = .37)
Poor (below FPL)	0.73 (0.61-0.88)^b^	0.63 (0.52-0.76)^b^	1.00 (0.61-1.65) (*P* = .99)	0.96 (0.53-1.74) (*P* = .90)
High-share insurance group				
Public insurance	1.03 (0.94-1.12) (*P* = .51)	0.98 (0.90-1.07) (*P* = .68)	0.78 (0.61-1.00) (*P* = .05)	1.19 (0.89-1.60) (*P* = .24)
Uninsured	1.55 (1.41-1.69)^b^	1.08 (0.99-1.20) (*P* = .10)	1.83 (1.37-2.44)	1.24 (0.88-1.75) (*P* = .21)

Abbreviations: aRR, adjusted rate ratio; CBG, census block group; EGS, emergency general surgery; FPL, federal poverty level.

^a^ All models adjusted for characteristics of CBG: median age; high-share racial and ethnic minority groups; median income; high-share insurance groups.

^b^ *P* < .001.

^c^ The category “other racial and ethnic minority groups” comprised Asian; American Indian, Alaska Native, or Pacific Islander; or 2 or more races and/or ethnicities.

Similar trends were observed for factors associated with low access to advanced-resource hospitals (Table 2). In micropolitan and rural areas, high-share Hispanic ethnicity and other racial and ethnic minority communities were also associated with low access, but with a greater aRR than for access to surgical hospitals overall. This was most pronounced in micropolitan communities, where high-share Hispanic CBGs had a nearly 3-fold risk of being a low-access area (aRR, 2.95; 95% CI, 2.38-3.66).

Sensitivity analysis with an examination of interactions between race and ethnicity and poverty revealed that in metropolitan areas and rural areas, poor and near-poor high-share other racial and ethnic minority CBGs had an increased risk of being in a low-access area for EGS hospitals overall compared with other racial and ethnic minority CBGs with income greater than 200% FPL. The same association was also pronounced for high-share Hispanic CBGs in micropolitan environments (eTable 5 in the Supplement).

## Discussion

In this cross-sectional study using advanced geospatial modeling techniques, we found substantial disparities in geospatial access to hospitals with emergency surgical capabilities across the US. Nearly 1 in 10 Americans experienced low spatial access to emergency surgical care, with 1 in 4 having low access to hospitals with advanced resources typically needed for life-threatening disease. This estimate exceeded those previously reported by studies using distance-based metrics alone. Rurality was significantly associated with low access with substantial variation observed in west and midwestern states compared with eastern and coastal regions. Small area analysis revealed insurance status to be a significant factor associated with spatial access to care across all settings, though high-share (>75th percentile) racial and ethnic minority status was further associated with low access to care in micropolitan and rural regions.

Unequal distribution of surgical care in the US is a recognized problem, but to date, the general understanding has been based on limited assessments of spatial access.^20,28,29^ To our knowledge, this was the first study to examine spatial access to emergency surgical care in the US at the CBG level using state-of-the-art E2SFCA methods. Evaluation of spatial access to EGS services using SPAR revealed greater population-level disparities in access than previously documented in prior studies using distance-based metrics. For example, Diaz et al^20^ evaluated access to inpatient surgical hospitals in 2015 using driving time and found 2% of the US population to be outside a 60-minute radius of any hospital with inpatient surgical services, and 8.4% were greater than 60 minutes from a major surgical hospital. Using metrics that incorporate population demand and measures of hospital capacity, this study raised concerns that using measures of travel distance or time alone might underestimate the proportion of the US population that experiences limitations in access to surgical care—using E2SFCA models, nearly 10% had low access to any surgical hospital, and 21% had low access to advanced-resource centers. This discordance aligned with our prior work^17^ assessing spatial access to EGS hospitals in California and the recent findings of Drake et al*,*^9^ who directly compared the E2SFCA with travel impedance measures of spatial access to buprenorphine prescribers and found that standard metrics (impedance measures and physician-to-population ratios) identified less than half of low-access census tracts compared with E2SFCA models.

A central finding of the present study was evidence of the intersectionality of race and ethnicity and rurality in spatial access to surgical care. In metropolitan areas, racial and ethnic minority communities tended to experience more favorable spatial access to surgical hospitals than predominantly White communities, consistent with other studies that have used both travel distance and time as primary metrics of access.^30^ In contrast, high-share Hispanic and other racial and ethnic minority group CBGs in micropolitan and rural areas were at substantially greater risk of being in a low-access area—a disparity that was magnified when access to advanced-resource hospitals was considered. This aligns with prior studies that have found inequities in travel distance to a range of hospital services, including emergency and trauma care, for rural patients who are members of racial and ethnic minority groups.^30,31^ The results of the present study further suggested that structural factors, including residential settlement patterns, contribute to inequities in access to surgical care. As others have noted, the intersection of race and ethnicity and rurality deserves more attention, and prior research has suggested that the disparities noted in health care access among rural racial and ethnic minority groups are often more severe than disparities noted in urban environments.^10,32,33,34^ The results of the present study underscored this point by identifying opposite patterns entirely in spatial access to surgical care for rural vs urban racial and ethnic minority groups. Rural America has become increasingly diverse over the past decade with 24% of rural Americans now identifying as “persons of color.”^35^ Policies guiding health systems development that do not consider the intersection of rurality and race and ethnicity will consequently be designed around urban populations and fail to address the needs of growing rural racial and ethnic minority populations. Analyses that separately address rural and urban populations are needed to ensure that rural health systems receive appropriate interventions for their specific community context to increase equitable access to care.

Ongoing trends of hospital mergers and closures have further affected spatial access for vulnerable communities in both urban and rural settings. Neighborhoods with high proportions of uninsured residents and Medicaid recipients have been disproportionately affected by hospital closures, which have led to longer travel distances even within urban regions.^36,37,38,39,40^ We observed similar patterns in this study, with uninsured or Medicaid status being consistent factors associated with low access to surgical care across urban, suburban, and rural regions. Together, the evidence suggests that payer mix is associated with hospital financial viability and can in turn explain marked inequities in spatial access to hospital care. Policies that address these factors exist, such as expansion of Medicaid and affordable insurance programs, but require meaningful metrics for assessing spatial access as well as commitment by policy makers to achieving equitable access to care.^30,41,42^

One challenge to ensuring adequate spatial access to care is the complexity inherent in measuring access itself. Prior studies of spatial access to surgical care have relied on either measure of travel impedance (eg, distance, time to nearest hospital), or provider-to-population ratios.^20,27,28,29^ Though intuitive, each holds serious limitations. Impedance measures can be problematic in urban areas where multiple hospitals exist within a similar distance, whereas, provider availability ratios are calculated within bordered units and do not account for variation in accessibility within geographic areas or patient border-crossing behavior.^8^ Gravity-based 2-step floating catchment area models, such as SPAR, address each of these concerns by estimating a provider-to-population ratio within a series of distance-based catchment areas and are not constrained by administrative borders. With regard to emergency surgical care, this method captures the essential element of distance to reach care, as well as hospital capacity to provide care relative to population needs. Nevertheless, E2SFCA models carry their own limitations. Most notably, it is a relative indicator based on the average value of an entire study region. Though useful in identifying disparity regions, it cannot be used to set minimum acceptable standards, as some portion of the population will always fall below a given threshold. We agree with the assertion made by Drake et al^9^ that E2SFCA models therefore should not replace more common measures of spatial access, but should be used alongside them in a set of spatial metrics to more accurately identify geographic areas with limited spatial access to surgical care that require targeted action in health system design. Such metrics require careful interpretation, as defaulting to increasing number of hospitals without attention to system effect can have negative outcomes with respect to patient care, as has been seen by proliferations of trauma centers in certain regions without clear attention to geographic or population needs.^43,44^

### Limitations

There are several limitations to this study. First, we used the AHA annual survey as a primary source to identify surgical hospitals, which is the largest source of information regarding hospital services in the US, but relies on administrative responses for accuracy of resource availability. We added additional hospitals not captured in national and state trauma databases, but nevertheless, the potential for hospital misclassification exists. We also used bed number as a measure of hospital capacity, which is the most commonly used measure in E2SFCA models of hospital access; however, it does not account for average hospital bed occupancy.^45,46,47^ Second, the presence of surgical capabilities did not necessarily equate to continuous service availability by surgeons, particularly at small rural hospitals that frequently experience workforce shortages and high staff turnover.^48,49,50,51^ The results of this study, therefore, provided an optimistic view of potential spatial access to surgical care in the US. In reality, disparities may have been even greater than reported in this study, and additional factors including patient insurance and existing patient-physician relationships may have been associated with hospital choice and realized access. Finally, there is no formal resource-level designation for hospitals that offer EGS care, unlike trauma center level designations.^52^ As such, this study’s definition of advanced-resource hospitals was intentionally broad and based on measures of both volume and proxy measures of resources, as has been used by others.

## Conclusions

In this cross-sectional study of spatial access to hospitals with emergency surgical capabilities using SPAR, we found that nearly 1 in 10 US residents experienced low access to care, and 1 in 4 had limited access to hospitals with the advanced resources necessary to treat complex surgical emergencies. Although insurance status was a factor associated with low access across all settings, racial and ethnic minority communities in micropolitan and rural areas experienced the greatest risk of limited access to surgical care. These findings support the use of E2SFCA models in identifying areas with low spatial access to surgical care and in guiding health system development.

## Notes

Supplement. **eFigure.** Hospital selection flowchart **eTable 1.** Hospital resources **eMethods.** Gravity-based spatial access model **eTable 2.** Regional variation in proportion of population living in CBGs with low access to any EGS-Capable Hospital **eTable 3.** Univariable models of factors associated with low-access CBGs **eTable 4.** Multinomial model of spatial access for all census block groups **eTable 5.** Interaction of race and ethnicity and poverty in predictors of census block group with low-access to any emergency general surgery capable hospital **eTable 6.** Comparison of logistic regression model adjusting for spatial autocorrelation with multinominal model risk of low vs. high spatial access
eReferences
