# Supplementary material for: Disparities in Spatial Access to Emergency Surgical Services in the US
Source: JAMA Health Forum. 2022 Oct 14;3(10):e223633. doi: 10.1001/jamahealthforum.2022.3633 (PMC9568808; doi:10.1001/jamahealthforum.2022.3633)
Supplement: Supplement. — eFigure. Hospital selection flowchart eTable 1. Hospital resources eMethods. Gravity-based spatial access model eTable 2. Regional variation in proportion of population living in CBGs with low access to any EGS-Capable Hospital eTable 3. Univariable models of factors associated with low-access CBGs eTable 4. Multinomial model of spatial access for all census block groups eTable 5. Interaction of race and ethnicity and poverty in predictors of census block group with low-access to any emergency general surgery capable hospital eTable 6. Comparison of logistic regression model adjusting for spatial autocorrelation with multinominal model risk of low vs. high spatial access eReferences [file jamahealthforum-e223633-s001.pdf]

Supplemental Online Content

McCrum ML, Wan N, Han J, Lizotte SL, Horns JJ. Disparities in spatial access to emergency surgical services in the US. *JAMA Health Forum.* 2022;3(10):e223633. doi:10.1001/jamahealthforum.2022.3633

eFigure. Hospital selection flowchart

eTable 1. Hospital resources

eMethods. Gravity-based spatial access model

eTable 2. Regional variation in proportion of population living in CBGs with low access to any EGS-Capable Hospital

eTable 3. Univariable models of factors associated with low-access CBGs

eTable 4. Multinomial model of spatial access for all census block groups

eTable 5. Interaction of race and ethnicity and poverty in predictors of census block group with low access to any emergency general surgery capable hospital

eTable 6. Comparison of logistic regression model adjusting for spatial autocorrelation with multinominal model risk of low vs. high spatial access

eReferences

This supplemental material has been provided by the authors to give readers additional information about their work.

© 2022 McCrum ML et al. *JAMA Health Forum.*

eFigure 1. Hospital selection flowchart

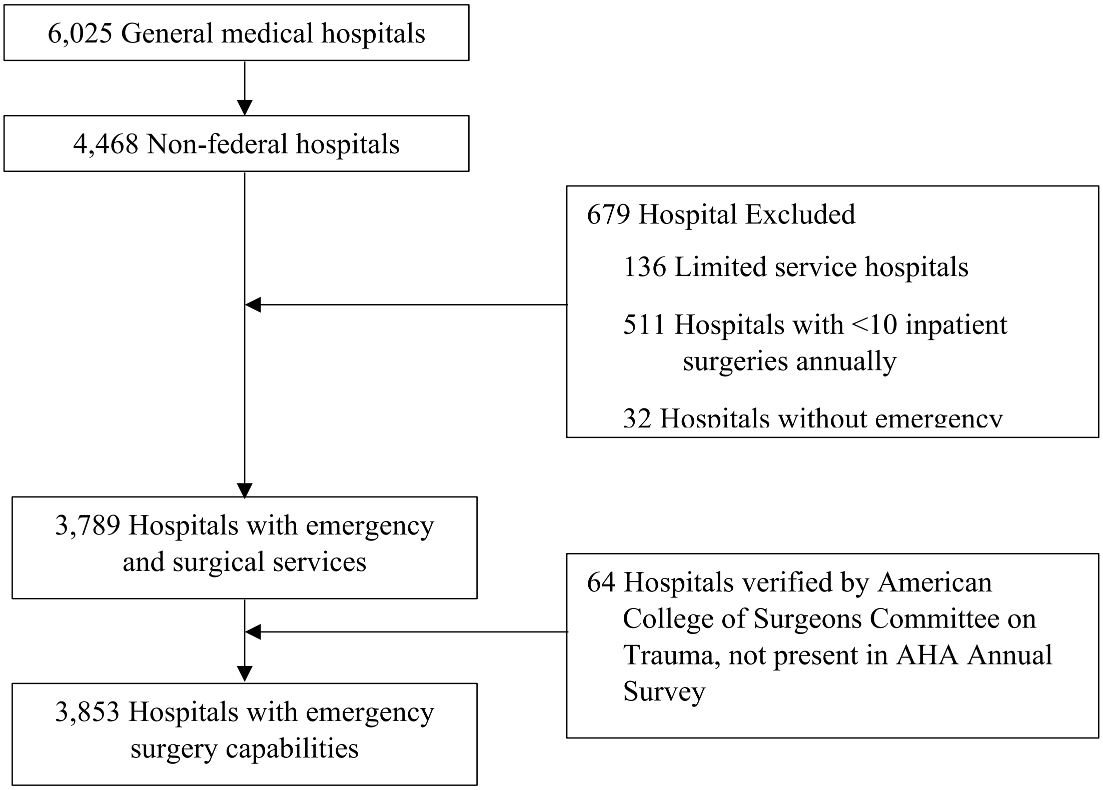

© 2022 McCrum ML et al. *JAMA Health Forum.*

eTable 1. Hospital resources

	Non-Advanced resource hospitals N=2787	Advanced resource hospitals N=1066
**Resources Considered in Definition of Advanced Resources**		
**CT Scanner** †	1,920 (98%)	1066 (100%)
**Ultrasound** †	1,881 (96%)	1066 (100%)
**ERCP**†	105 (11%)	1066 (100%)
**Medical/Surgical Intensive Care Unit Beds >5** †	382 (19.5%)	1066 (100%)
**Medical/Surgical ICU Beds** Median [Interquartile Range]	3 [0-6]	17 [11-28]
**Trauma Level**		
**1 or 2**	0	570 (53%)
**3 or 4**	498 (18%)	301 (28%)
**Non-trauma**	2289 (82%)	195 (18%)
**Number of annual inpatient surgeries** Median [Interquartile Range]	360 [137-1002]	2800 [1460-5311]
**Additional Resources**		
**Total bed number** Median [Interquartile Range]	53 [25-120]	388 [146-388]]
**Medical School Affiliation**	724 (16%)	765 (48%) (43.0%)

*All values shown are N (%) unless otherwise noted
† N= 3,026
ERCP = Endoscopic Retrograde Cholangiopancreatography; ICU=Intensive Care Unit

© 2022 McCrum ML et al. *JAMA Health Forum.*

eMethods: Gravity-based spatial access model

Realizing the limitations of both travel impedance (cost) measures and provider-population ratios in modeling spatial access to healthcare, researchers have adopted gravity models to account for the complicated interactions among healthcare supply, population demand for healthcare, and travel impedance between population locations and healthcare sites.[1–4] Gravity-based spatial access models estimate spatial access to medical services based on the law of gravitation.[5] Specifically, gravity models assume a population site's spatial access to a medical site decreases with the increase of travel distance to that medical site. A distance impedance function, *f(d)*, is generally used to model the influence of travel distance *d* on the spatial access.

One of the most commonly used and widely validated gravity models is the enhanced 2-step floating catchment area (E2SFCA) method.[2,3,6–8] Given *m* population sites (e.g., CBG centroids) and *n* medical sites (e.g., hospitals) in a study area, E2SFCA works in two steps. The first step calculates the supply-demand ratio of each medical site, *j*. Specifically, it generates a 60-minute driving zone (also called a catchment area) around *j*, divides the catchment into four contiguous zones based on predefined driving time intervals (e.g., 0-10 min, 10-20 min, 20-30 min, 30-60), searches all population sites within each zone, and calculates the supply-demand ratio for *j* by

$$R_j = \frac{S_j}{\sum_{j\in(d_{kj}\in D_r)} P_k W_r} = \frac{S_j}{\sum_{j\in(d_{kj}\in D_1)} P_k W_1 + \sum_{j\in(d_{kj}\in D_2)} P_k W_2 + \sum_{j\in(d_{kj}\in D_3)} P_k W_3 + \sum_{j\in(d_{kj}\in D_4)} P_k W_4}$$

where S_j is the medical capacity (estimated by number of inpatient beds) of medical site *j*, P_k is the population size of the *k*th population site within the catchment, d_{kj} is the travel cost between *j* and *k*, D_r is the *r*th sub-zone, and W_r is a distance-based weight for D_r. Following previous studies[1,3,4], we used the Gaussian function (i.e., $f(d) = e^{-d^2/\beta}$ where d represents a distance and β represents an impedance parameter) to calculate W_r. More details on the Gaussian function and the calculation of W_r can be found in Wan et al. 2012[4].

The second step of E2SFCA is to calculate a Spatial Access Index (SPAI) for each population site *i*. Specifically, a 60-min catchment and four driving zones (i.e., 0-10 min, 10-20 min, 20-30 min, 30-60 min) are generated for *i*, following the same procedures in the first step. Then it summarizes the supply-demand ratios of all medical sites within the catchment using the following formula:

$$A_i = \sum_{k\in(d_{ik}\in D_r)} R_k W_r = \sum_{k\in(d_{ik}\in D_1)} R_k W_1 + \sum_{k\in(d_{ik}\in D_2)} R_k W_2 + \sum_{k\in(d_{ik}\in D_3)} R_k W_3 + \sum_{k\in(d_{ik}\in D_4)} R_k W_4$$

where A_i is the SPAI for *i*, R_k is the supply-to-demand ratio (calculated in step 1) of medical site *k* that falls inside the catchment of *i*, and d_{ik} is the travel time between *k* and *i*. W_r is the same distance-based weight calculated in step 1.

The E2SFCA implements the idea of gravity assumption, as a shorter distance denotes a higher population demand for a hospital (realized by function *f(d)* in step 1) and better spatial access for a population site (realized by function *f(d)* in the second demand). Therefore, a higher A_i denotes a better spatial access, and vice versa.

The above mentioned E2SFCA method will be used to examine spatial access to emergency surgical services (for both all hospitals with emergency surgical capabilities and advanced-resource centers) in the United States in this study. Specifically, the population size of each CBG is used to approximate demand and number of inpatient beds at each hospital is used to represent the relative capacity of each hospital. CBG population size is the most direct

measure of population demand, as CBG-level estimates of EGS disease are not available. Hospital bed number is commonly used as a marker of facility size and capacity and is frequently used in health care research and planning. Both CBG population size and number of hospital beds are standard measures used in other studies of spatial access to hospital care using E2SCA models.[9–11]

To minimize the influence of the infamous distance impedance problem (i.e., the selection of the impedance parameter β could influence the spatial access results), we used a weighted spatial access index, Spatial Access Ratio (SPAR)[4], to represent the eventual result. SPAR for a population site is calculated as the ratio between that population site's SPAI and the average of SPAI among all population sites in the study area. The higher the SPAR, the better the spatial access. And SPAR values great than one means better-than-state-average spatial access, and vice versa. SPAR has been proved effective in overcoming the distance impedance problem in multiple studies and has been used to explore spatial access to a variety of healthcare services[1,12–14].

© 2022 McCrum ML et al. *JAMA Health Forum.*

eTable 2: Regional variation in proportion of population living in CBGs with low access to any EGS-capable hospital

		Population in Low-Access CBG (N, %)	
Region	Total Population	Any EGS-Capable Hospital	Advanced-Resource Hospital
Northeast	54,742,785	2,498,809<br>4.61%	8,711,221<br>15.9%
Midwest	66,885,126	6,992,348<br>10.5%	21,847,527<br>32.7%
South	118,770,525	13,259,211<br>11.2%	34,076,915<br>28.7%
West	74,678,812	7,583,587<br>10.2%	16,705,812<br>22.4%

CBG=Census Block Group; EGS=Emergency General Surgery

© 2022 McCrum ML et al. *JAMA Health Forum.*

eTable 3. Univariable models of factors associated with low-access CBGs

a. Metropolitan

	All EGS Hospitals		Advanced Clinical Resource Hospitals	
	Low Access RR (95% CI)	Medium Access RR (95% CI)	Low Access RR (95% CI)	Medium Access RR (95% CI)
**Median Age**	1.05 (1.05-1.05)*	1.00 (1.00 – 1.00)	1.01 (1.01-1.02)*	1.00 (1.00-1.01)*
**High-share Minority**				
Black	0.27 (0.25-0.28)*	0.36 (0.35-0.37)*	0.38 (0.37-0.39)*	0.37 (0.36-0.38)*
Hispanic	0.53 (0.51-0.56)*	1.28* (1.25-1.30)	0.58 (0.56-0.60)*	1.20 (1.17-1.23)*
Other Racial and Ethnic Minority Groups	0.30 (0.28-0.32)*	1.29 (1.26-1.32)*	0.40 (0.39-0.42)*	1.42 (1.40-1.46)*
**Median Income**				
Non-poor (>200% FPL)	1 [Reference]		1 [Reference]	
Near-poor (100-200% FPL)	0.73 (0.69-0.77)*	0.44 (0.43-0.45)*	1.03 (0.99-1.07)	0.45 (0.44-0.46)*
Poor: Below FPL	0.23 (0.19-0.27)*	0.21 (0.20-0.22)*	0.58 (0.54-0.63)*	0.23 (0.21-0.24)*
**High-share Insurance**				
Public Insurance	1.07 (1.01-1.14)^	0.55 (0.53-0.56)*	1.17 (1.12-1.22)*	0.58 (0.57-0.60)*
Uninsured	1.41 (1.33-1.49)*	0.64 (0.63-0.66)*	1.12 (1.07-1.16)*	0.61 (0.60-0.63)*

CBG=Census Block Group; EGS= Emergency General Surgery; RR= rate ratio; FPL=Federal Poverty Limit

^p<0.05; *p<0.001

© 2022 McCrum ML et al. *JAMA Health Forum.*

b. Micropolitan

	All EGS Hospitals		Advanced Clinical Resource Hospitals	
	Low Access RR (95% CI)	Medium Access RR (95% CI)	Low Access RR (95% CI)	Medium Access RR (95% CI)
**Median Age**	1.03 (1.03-1.04)*	1.01 (1.01-1.01)*	1.01 (1.00-1.02)*	1.00 (0.99-1.00)
**High-share Minority**				
Black	0.42 (0.47-0.52)*	0.46 (0.42-0.49)*	0.47 (0.42-0.53)*	0.48 (0.40-0.57)*
Hispanic	1.21 (1.08-1.37)*	1.34 (1.23-1.46)*	3.02 (2.45-3.72)*	2.95 (2.31-3.78)*
Other Racial and Ethnic Minority Groups	1.06 (0.94-1.20)	0.81 (0.74-0.89)*	1.77 (1.47-2.13)*	1.17 (0.90-1.50)
**Median Income**				
Non-poor (>200% FPL)	1 [Reference]		1 [Reference]	
Near-poor (100-200% FPL)	0.71 (0.65-0.77)*	0.74 (0.70-0.79)*	1.09 (0.99-1.22)	0.92 (0.79-1.06)
Poor: Below FPL	0.33 (0.27-0.39)*	0.42 (0.37-0.47)*	0.72 (0.60-0.86)*	1.06 (0.63-1.78)
**High-share Insurance**				
Public Insurance	10.97 (0.89-1.06)	0.77 (0.72-0.82)*	1.25 (1.11-1.39)*	1.09 (0.94-1.27)
Uninsured	1.02 (0.93-1.12)	1.00 (0.92-1.08)	1.17 (1.04-1.31)^	1.10 (0.94-1.29)

CBG=Census Block Group; EGS= Emergency General Surgery; RR= rate ratio; FPL=Federal Poverty Limit

^p<0.05; *p<0.001

© 2022 McCrum ML et al. *JAMA Health Forum.*

c. Rural

	All EGS Hospitals		Advanced Clinical Resource Hospitals	
	Low Access RR (95% CI)	Medium Access RR (95% CI)	Low Access RR (95% CI)	Medium Access RR (95% CI)
**Median Age**	1.02 (1.02-1.03)*	1.01 (1.00-1.01)^	1.03 (1.02-1.04)*	1.00 (0.99-1.01)
**High-share Minority**				
Black	0.61 (0.55-0.67)*	0.83 (0.75-0.92)*	0.54 (0.41-0.69)*	0.83 (0.60-1.13)
Hispanic	1.73 (1.52-1.98)*	1.21 (1.05-1.38)^	1.74 (1.12-2.71)^	1.52 (0.91-2.53)
Other Racial and Ethnic Minority Groups	1.29 (1.15-1.45)*	0.87 (0.76-0.98)^	11.73 (1.13-2.65)^	1.22 (0.74-2.01)
**Median Income**				
Non-poor (>200% FPL)	1 [Reference]		1 [Reference]	
Near-poor (100-200% FPL)	0.88 (0.82-0.95)*		0.98 (0.79-1.22)	0.93 (0.72-1.22)
Poor: Below FPL	0.69 (0.59-0.81)*	0.60 (0.51-0.70)*	0.73 (0.47-1.13)	1.06 (0.63-1.78)
**High-share Insurance**		0.86 (0.80-0.93)*		
Public Insurance	0.91 (0.84-0.98)^	0.88 (0.81-0.95)*	0.75 (0.61-0.94)^	1.12 (0.87-1.46)
Uninsured	1.40 (1.29-1.52)*	1.00 (0.92-1.08)	1.70 (1.30-2.22)*	1.22 (0.89-1.68)

CBG=Census Block Group; EGS= Emergency General Surgery; RR= rate ratio; FPL=Federal Poverty Limit

^p<0.05; *p<0.001

© 2022 McCrum ML et al. *JAMA Health Forum.*

eTable 4. Multinomial model of spatial access for all census block groups

a) All EGS Hospitals

	**Low Access**	**Medium Access**
	aRR (95% CI)	aRR (95% CI)
**Median Age**	1.03 (1.03-1.03)*	1.01 (1.00-1.01)*
**High-share Minority**		
Black	0.27 (0.26-0.28)*	0.46 (0.45-0.47)*
Hispanic	0.57 (0.55-0.60)*	1.42 (1.38-1.45)*
Other Racial and Ethnic Minority Groups	0.42 (0.40-0.44)*	1.06 (1.03-1.08)*
**Median Income**		
Non-poor (>200% FPL)	1 [Reference]	1 [Reference]
Poor: Below FPL	0.52 (0.48-0.57)*	0.39 (0.37-0.40)*
Near-poor (100-200% FPL)	1.04 (1.00-1.08)	0.64 (0.63-0.66)*
**High-share Insurance**		
Public Insurance	1.21 (1.16-1.25)*	0.95 (0.93-0.98)*
Uninsured	1.58 (1.52-1.64)*	0.90 (0.87-0.92)

^p<0.05; *p<0.001

© 2022 McCrum ML et al. *JAMA Health Forum.*

b) Advanced Resource Hospitals

	Low Access	Medium Access
	aRR (95% CI)	aRR (95% CI)
**Median Age**	1.02 (1.02-1.02)*	1.00 (1.00-1.01)*
**High-share Minority**		
Black	0.28 (0.27-0.28)*	0.47 (0.46-0.49)*
Hispanic	0.42 (0.41-0.43)*	1.42 (1.39-1.46)*
Other Racial and Ethnic Minority Groups	0.39 (0.38-0.41)*	1.26 (1.23-1.29)*
**Median Income**		
Non-poor (>200% FPL)	1 [Reference]	1 [Reference]
Poor: Below FPL	1.18 (1.12-1.24)*	0.38 (0.35-0.40)*
Near-poor (100-200% FPL)	1.76 (1.71-1.80)*	0.59 (0.57-0.60)*
**High-share Insurance**		
Public Insurance	1.25 (1.21-1.28)*	0.98 (0.95-1.01)
Uninsured	1.29 (1.25-1.32)*	0.81 (0.79-0.84)*

^p<0.05; *p<0.001

© 2022 McCrum ML et al. *JAMA Health Forum.*

eTable 5. Interaction of race and ethnicity and poverty in predictors of census block group with low-access to any emergency general surgery capable hospital*

	aRR	95% CI	p-value
**Metropolitan**			
Black Q4: Below FPL	0.39	0.30 - 0.55	< 0.001
Black Q4: Near-poor (>200% FPL)	0.82	0.73 - 0.93	0.001
Hispanic Q4: Below FPL	0.64	0.44 - 0.91	0.01
Hispanic Q4: Near-poor (>200% FPL)	0.52	0.47 - 0.59	< 0.001
Other Minority Q4: Below FPL	2.42	1.61 - 3.63	< 0.001
Other Minority Q4: Near-poor (>200% FPL)	1.30	1.12 - 1.52	0.001
**Micropolitan**			
Black Q4: Below FPL	1.47	0.93 - 2.30	0.10
Black Q4: Near-poor (>200% FPL)	0.96	0.71 – 1.28	0.76
Hispanic Q4: Below FPL	1.83	1.12 – 2.98	0.02
Hispanic Q4: Near-poor (>200% FPL)	1.12	0.94 – 1.57	0.13
Other Minority Q4: Below FPL	0.70	0.39 – 1.26	0.24
Other Minority Q4: Near-poor (>200% FPL)	0.91	0.71 – 1.18	0.47
**Rural**			
Black Q4: Below FPL	1.06	0.71 – 1.59	0.80
Black Q4: Near-poor (>200% FPL)	1.05	0.79 – 1.40	0.73
Hispanic Q4: Below FPL	1.11	0.62 – 1.97	0.73
Hispanic Q4: Near-poor (>200% FPL)	0.79	0.59 – 1.05	0.10
Other Minority Q4: Below FPL	3.71	2.21 – 6.37	< 0.001
Other Minority Q4: Near-poor (>200% FPL)	1.57	1.22 – 2.03	0.001

*All models adjusted for Age, High-Share Race/Ethnicity groups, Median Income, High-share Insurance groups

© 2022 McCrum ML et al. *JAMA Health Forum.*

eTable 6. Comparison of logistic regression model adjusting for spatial autocorrelation with multinominal model risk of low vs. high spatial access±

	**Metropolitan**		**Micropolitan**		**Rural**	
	**Logistic Regression** Model aOR (95% CI)	**Multinomial** Model aRR (95% CI)	**Logistic Regression** Model aOR (95% CI)	**Multinomial** Model aRR (95% CI)	**Logistic Regression** Model aOR (95% CI)	**Multinomial** Model aRR (95% CI)
**Median Age**	1.03 (1.03-1.03)*	1.03 (1.03-1.03)*	1.03 (1.03-04)*	1.03 (1.03-1.04)*	1.03 (1.02-1.03)*	1.03 (1.03-1.03)*
**High-share Minority**						
Black	0.31 (0.30-0.33)*	0.33 (0.31-0.35)*	0.51 (0.45 – 0.57)*	0.51 (0.44-0.57)*	0.66 (0.59-0.74)*	0.64 (0.58-0.72)*
Hispanic	0.58 (0.54-0.62)*	0.63 (0.60-0.68)*	1.34 (1.18-1.53)*	1.35 (1.19-1.54)*	1.80 (1.57-2.07)*	1.80 (1.57-2.01)*
Other Racial and Ethnic Minority Groups	0.29 (0.27-0.31)*	0.30 (0.28-0.32)*	1.10 (0.97-1.25)	1.12 (0.99-1.28)	1.33 (1.17 – 1.50)*	1.31 (1.16-1.48)*
**Median Income**						
Non-poor (>200% FPL)	1 [Reference]		1 [Reference]		1 [Reference]	
Near-poor (100-200% FPL)	0.74 (0.70-0.78)*	0.73 (0.69-0.77)*	0.68 (0.61-0.75)*	0.70 (0.63-0.77)*	0.84 (0.77-0.91)*	0.84 (0.78-0.92)*
Poor: Below FPL	0.23 (0.20-0.28)*	0.23 (0.19-0.27)*	0.36 (0.29-0.45)*	0.38 (0.31-0.47*)	0.73 (0.61-0.88)*	0.73 (0.61-0.88)*
**High-share Insurance**						
Public Insurance	1.09 (1.03-1.16)*	1.07 (1.01-1.14)^	1.36 (1.23-1.51)*	1.34 (1.22-1.48)^	1.03 (0.94-1.12)	1.03 (0.94-1.12)
Uninsured	1.40 (1.33-1.49)*	1.41 (1.33-1.49)*	1.45 (1.31-1.61)*	1.40 (1.26-1.55)*	1.53 (1.40-1.68)*	1.55 (1.41-1.69)*

±Logistic regression model adjusts for spatial autocorrelation using an exponential spatial covariance structure, where covariance between observations is based on Euclidian distance between centroids

^p<0.05; *p<0.001

© 2022 McCrum ML et al. *JAMA Health Forum.*

eReferences

1. Wan N, Zou B, Sternberg T. A three-step floating catchment area method for analyzing spatial access to health services. *Int J Geogr Inf Sci*. 2012;26(6):1073-1089. doi:10.1080/13658816.2011.624987

2. Guagliardo MF. Spatial accessibility of primary care: concepts, methods and challenges. *Int J Health Geogr*. 2004;3(1):3. doi:10.1186/1476-072X-3-3

3. Luo W, Qi Y. An enhanced two-step floating catchment area (E2SFCA) method for measuring spatial accessibility to primary care physicians. *Health Place*. 2009;15(4):1100-1107. doi:10.1016/j.healthplace.2009.06.002

4. Wan N, Zhan FB, Zou B, Chow E. A relative spatial access assessment approach for analyzing potential spatial access to colorectal cancer services in Texas. *Appl Geogr*. 2012;32(2):291-299. doi:10.1016/j.apgeog.2011.05.001

5. Joseph AE, Bantock PR. Measuring potential physical accessibility to general practitioners in rural areas: A method and case study. *Soc Sci Med*. 1982;16(1):85-90. doi:10.1016/0277-9536(82)90428-2

6. McGrail MR. Spatial accessibility of primary health care utilising the two step floating catchment area method: an assessment of recent improvements. *Int J Health Geogr*. 2012;11(1):50. doi:10.1186/1476-072X-11-50

7. Drake C, Nagy D, Nguyen T, et al. A comparison of methods for measuring spatial access to health care. *Health Serv Res*. 2021;56(5):777-787. doi:10.1111/1475-6773.13700

8. Naylor KB, Tootoo J, Yakusheva O, Shipman SA, Bynum JPW, Davis MA. Geographic variation in spatial accessibility of U.S. healthcare providers. *PLoS ONE*. 2019;14(4):e0215016. doi:10.1371/journal.pone.0215016

9. Deguen FG and MJ and S. Measuring hospital spatial accessibility using the enhanced two-step floating catchment area method to assess the impact of spatial accessibility to hospital and non-hospital care on the length of hospital stay | EndNote Click. Accessed August 4, 2022. https://click.endnote.com/viewer?doi=10.1186%2Fs12913-021-07046-3&token=WzI1ODYwOTQsIjEwLjExODYvczEyOTEzLTAyMS0wNzA0Ni0zIl0.OkrSFBFccDSEeRPVom3IpvrLzRo

10. al JYK et. Rapidly measuring spatial accessibility of COVID-19 healthcare resources: a case study of Illinois, USA | EndNote Click. Accessed August 4, 2022. https://click.endnote.com/viewer?doi=10.1186%2Fs12942-020-00229-x&token=WzI1ODYwOTQsIjEwLjExODYvczEyOTQyLTAyMC0wMDIyOS14Il0.N17FQbznwyjNz-wdDlRAPkCL9ss

11. Bauer J, Klingelhöfer D, Maier W, Schwettmann L, Groneberg DA. Prediction of hospital visits for the general inpatient care using floating catchment area methods: a reconceptualization of spatial accessibility. *Int J Health Geogr*. 2020;19(1):29. doi:10.1186/s12942-020-00223-3

12. Wan N, Zhan FB, Zou B, Wilson JG. Spatial Access to Health Care Services and Disparities in Colorectal Cancer Stage at Diagnosis in Texas. *Prof Geogr*. 2013;65(3):527-541. doi:10.1080/00330124.2012.700502

13. Wan N, McCrum M, Han J, et al. Measuring spatial access to emergency general surgery services: does the method matter? *Health Serv Outcomes Res Methodol*. Published online 2021. doi:10.1007/s10742-021-00254-8

14. McCrum ML, Wan N, Lizotte SL, Han J, Varghese T, Nirula R. Use of the spatial access ratio to measure geospatial access to emergency general surgery services in California. *J Trauma Acute Care Surg*. 2021;90(5):853-860. doi:10.1097/TA.0000000000003087

© 2022 McCrum ML et al. *JAMA Health Forum.*
